# Development of mouse model for oral allergy syndrome to identify IgE cross-reactive pollen and food allergens: ragweed pollen cross-reacts with fennel and black pepper

**DOI:** 10.3389/fimmu.2022.945222

**Published:** 2022-07-25

**Authors:** Anna Kamei, Kumi Izawa, Tomoaki Ando, Ayako Kaitani, Risa Yamamoto, Akie Maehara, Takuma Ide, Hiromichi Yamada, Mayuki Kojima, Hexing Wang, Koji Tokushige, Nobuhiro Nakano, Toshiaki Shimizu, Hideoki Ogawa, Ko Okumura, Jiro Kitaura

**Affiliations:** ^1^ Atopy (Allergy) Research Center, Juntendo University Graduate School of Medicine, Tokyo, Japan; ^2^ Department of Science of Allergy and Inflammation, Juntendo University Graduate School of Medicine, Tokyo, Japan; ^3^ Department of Otorhinolaryngology, Juntendo University Graduate School of Medicine, Tokyo, Japan; ^4^ Department of Pediatrics and Adolescent Medicine, Juntendo University Graduate School of Medicine, Tokyo, Japan

**Keywords:** oral allergy syndrome (OAS), mouse model, IgE, cross-reactivity, pollen, food, allergen

## Abstract

Oral allergy syndrome (OAS) is an IgE-mediated immediate food allergy that is localized to the oral mucosa. Pollen food allergy syndrome (PFAS), a pollinosis-associated OAS, is caused by cross-reactivity between food and pollen allergens. However, we need to more precisely understand the underlying pathogenesis of OAS/PFAS. In the present study, we developed a method to comprehensively identify cross-reactive allergens by using murine model of OAS and protein microarray technology. We focused on lip angioedema, which is one of the most common symptoms of OAS, and confirmed that mast cells reside in the tissues inside the lower lip of the mice. Interestingly, when the food allergen ovalbumin (OVA) was injected inside the lower lip of mice with high levels of OVA-specific IgE followed by an intravenous injection of the Evans blue dye, we found immediate dye extravasation in the skin of the neck in a mast cell-dependent manner. In addition, the degree of mast cell degranulation in the oral cavity, reflecting the severity of oral allergic responses, can be estimated by measuring the amount of extravasated dye in the skin. Therefore, we used this model of OAS to examine IgE cross-reactive allergens *in vivo*. Protein microarray analysis showed that serum IgE from mice intraperitoneally sensitized with ragweed pollen, one of the major pollens causing pollinosis, bound highly to protein extracts from several edible plants including black peppercorn and fennel. We confirmed that the levels of black pepper-specific IgE and fennel-specific IgE were significantly higher in the serum from ragweed pollen-sensitized mice than in the serum from non-sensitized control mice. Importantly, analysis of murine model of OAS showed that the injection of black pepper or fennel extract induced apparent oral allergic responses in ragweed pollen-sensitized mice. These results indicate IgE cross-reactivity of ragweed pollen with black pepper and fennel. In conclusion, we developed mouse model of OAS to identify IgE cross-reactive pollen and food allergens, which will help understand the pathogenesis of OAS/PFAS.

## Introduction

Food allergy is defined as a phenomenon in which adverse reactions are caused by allergen-specific immunological mechanisms following exposure to a given food (1–5). Oral allergy syndrome (OAS) involves the rapid onset of symptoms induced by food allergens in the oral and pharyngeal mucosa, including itching and/or angioedema of the lip, tongue, palate, and throat. In many cases, OAS is an IgE-mediated food allergy that can be induced by any allergen in animal foods such as eggs and milk or plant foods such as fresh fruits and vegetables (6–9). Pollinosis-associated OAS is called pollen food allergy syndrome (PFAS), in which certain fruits and vegetables show cross-reactivity with pollen such as birch pollen (10–13). Food allergens are classified into class I and class II types. Class I food allergens (e.g., milk) are oral allergens that cause sensitization through the gastrointestinal tract, whereas class II food allergens are highly homologous to aeroallergens that cause sensitization through the respiratory tract. Class II food allergens (e.g., vegetables and fruits) are generally heat-labile and easy-to-digest proteins that are structurally similar to pollen homologs. Profilins and Bet v 1-like proteins are class II food allergens that are responsible for OAS. After sensitization with specific pollen, ingestion of certain fruits or vegetables that cross-react with pollen induces IgE-mediated mast cell degranulation in the oral cavity of patients with PFAS (14–18). It has been reported that birch pollen cross-reacts with *Rosaceae* (e.g., apple, pear, and peach) (19–21), mugwort pollen cross-reacts with *Apiaceae* (e.g., celery) (22, 23) or with spice, and ragweed pollen cross-reacts with *Cucurbitaceae* (e.g., melon) (24–27) or with *Musaceae* (e.g., banana) (28, 29) to induce PFAS (30–33).

The spectrum of PFAS symptoms ranges from oropharyngeal symptoms to anaphylactic shock. The reported prevalence of PFAS varies between 2% and 70% according to geographical location. PFAS is generally diagnosed by taking a history from the relevant patients, performing a skin prick test, and measuring serum levels of allergen-specific IgE antibodies (6–17). However, the molecular mechanisms by which aeroallergens show cross-reactivity with food allergens are elusive. In this study, we used murine model of sensitization to ragweed pollen and protein microarray technology (34) to develop methods for the comprehensive identification of foods that may cross-react with ragweed pollen. We identified black pepper and fennel as foods that may cross-react with ragweed pollen using this method and demonstrated IgE cross-reactivity of ragweed pollen with black pepper or fennel using murine model of OAS. Although further examination is required to clarify the clinical significance and molecular mechanisms underlying the cross-reactivity between them, analyses using this newly developed method may clarify the pathogenesis of unexplained cases of OAS/PFAS.

## Material and methods

### Mice

All procedures were approved by the Institutional Review Committee of Juntendo University (approval numbers 2020128 and 2020136). Wild-type (WT) and *Kit^W-sh/W-sh^* (mast cell-deficient) mice with a BALB/c background (aged 8‐12 weeks) were used as murine models for OAS (35, 36).

### Antibodies and other reagents

The following antibodies were used and were purchased from BioLegend (San Diego, CA): allophycocyanin (APC)/cyanine 7 (Cy7)-conjugated anti-mouse CD45, phycoerythrin/Cy7-conjugated anti-mouse FcεRIα (MAR-1), and APC-conjugated anti-mouse c-Kit. Ragweed pollen was purchased from PolyScience (Niles, IL). 4’,6-Diamidino-2-phenylindole Dihydrochloride (DAPI) was purchased from Dojindo (Kumamoto, Japan). OVA (grade V) was purchased from Sigma-Aldrich (St Louis, MO). Alum was purchased from Thermo Fisher Scientific Inc. (Waltham, MA). Compound 48/80 was purchased from Sigma-Aldrich (St Louis, MO). To prepare fennel extracts, commercially available fennel from Japan (Fruits and vegetables of Otsuru, Fukuoka, Japan) mixed with phosphate-buffered saline (PBS) was ground using a food processor. To prepare the black pepper extracts, commercially available black pepper from Malaysia (S&B Foods Inc., Tokyo, Japan) was ground in a mortar and mixed with PBS. The mixtures were then filtrated into the tube before centrifugation at 20,000g for 10 min. The supernatants were collected and subjected to BCA protein assay (Fujifilm, Tokyo, Japan) to measure protein concentration.

### Allergenic protein microarray

Serum from Balb/c mice intraperitoneally injected with 100 μg ragweed pollen plus 2 mg alum or with 2 mg alum alone as a control six times at 1-week intervals was incubated on microarray plates coated with crude allergenic protein extracts from various plants, animals, processed foods, and microorganisms (total 1178 types), including vegetables, fruits, fishes, shellfishes, meats, eggs, cheeses, yogurts, insects, ticks, parasites, bacteria, and fungi (Fukushima Translational Research Project, Fukushima, Japan). After Alexa Fluor 647-conjugated anti-mouse IgE antibody was added to the wells, microarray plates were scanned with a GenePix 4000 B scanner (Molecular Devices, San Jose, CA) and fluorescence intensity was measured. Allergenic protein microarray analysis was performed (Fukushima Translational Research Project, Fukushima, Japan) (34). The relative binding intensity of serum IgE to the respective protein extracts was estimated by subtracting the IgE-binding intensity of serum from mice injected with alum alone from IgE-binding intensity of serum from mice injected with ragweed pollen plus alum.

### Measurements for total IgE, ragweed, Fennel, or black pepper-specific IgE, or cytokines using enzyme-linked immunosorbent assay

ELISA kits for total IgE (eBioscience, San Diego, CA), and interleukin-4 (IL-4) and IL-13 (R&D Systems, Minneapolis, MN) were used. Ragweed, fennel, or black pepper-specific IgE was determined using luminescence ELISA as previously described (35, 36). Briefly, ragweed pollen, fennel, or black pepper extract-coated ELISA plates were blocked before adding serial dilutions of serum samples before washing the wells. After biotinylated anti-IgE Ab (R35-118) (BD Pharmingen, San Diego, CA) was added, the plates were incubated and washed. Streptavidin-horseradish peroxidase was added, the plates were incubated and washed. Then, 3,3’,5,5’-tetramethylbenzidine substrate solution and stop solution were added (BD Biosciences, San Jose, CA), before measuring the absorbance at a wavelength of 450 nm wavelength using a microplate reader.

### Murine model of OAS

The mice were injected with 500 ng compound 48/80 or PBS as a control inside the lower lip just immediately before the intravenous injection of the Evans blue dye. Alternatively, 14 days after intraperitoneal injection of 100 μg OVA plus 2 mg alum twice with a 2-week interval, the mice were given an injection of 2 μg OVA or PBS as a control inside the lower lip right before the intravenous injection of the Evans blue dye. Seven days after intraperitoneal injection of 100 μg ragweed pollen plus 2 mg alum six times with a 1-week interval, the mice were given an injection of 4 μg black pepper extract, 4 μg fennel extract, or PBS as a control inside the lower lip immediately before the intravenous injection of the Evans blue dye. Three days after intranasal injection of 1 mg ragweed pollen for five consecutive days in the first and second week following intraperitoneal injection of 100 μg ragweed pollen plus 2 mg alum three times with a 1-week interval (37–40), the mice were administered an injection of 4 μg fennel extract or PBS as a control inside the lower lip right before the intravenous injection of 0. 2 mL of 0.1% Evans blue dye. The skin of the neck removed 30 min after dye injection was cut into small pieces that were put into 0.3 mL of 1N KOH and left overnight at 37°C with shaking. On the next day, 0.15 mL of 1N phosphoric acid and 0.39 mL of acetone was added to the solution before mixing by inverting. After centrifugation at 700g for 15 min, 0.2 mL of the supernatant was added into each well of 96-well microplate. The amount of extravasated dye was evaluated by measuring the absorbance at a wavelength of 620 nm on 96-well microplate luminometer, as previously described (36, 41–44).

### Histological analyses

Histological analyses were performed as previously described (35, 36). Sections of the tissue in the inside of the lower lip of Balb/c mice were stained with both toluidine blue or chloroacetate esterase.

### Flow cytometry

Suspension cells were prepared after mincing and digesting the tissue in the inside of the lower tip with 2 mg/ml collagenase type I (FUJIFILM, Tokyo, Japan) and 0.1 mg/ml DNase I (Roche Diagnostic, Mannheim, Germany) for 1 h at 37 °C. Stained cells were analyzed on a FACSVerse flow cytometer (BD Biosciences, San Jose, CA) and data were analyzed using FlowJo software (Tree Star), as previously described (35, 36).

### *In vitro* analysis of the Th2 response

Single-cell suspensions of cervical lymph node (LN) cells (2 × 10^6^) from mice sensitized intraperitoneally and intranasally with ragweed pollen or from non-sensitized mice were cultured in the presence of 20 μg/mL ragweed pollen, black pepper, or fennel extract or PBS as a control for 4 days to measure the levels of cytokines IL-4 or IL-13 in culture supernatants (35).

### Statistical analyses

**Figure 1:**
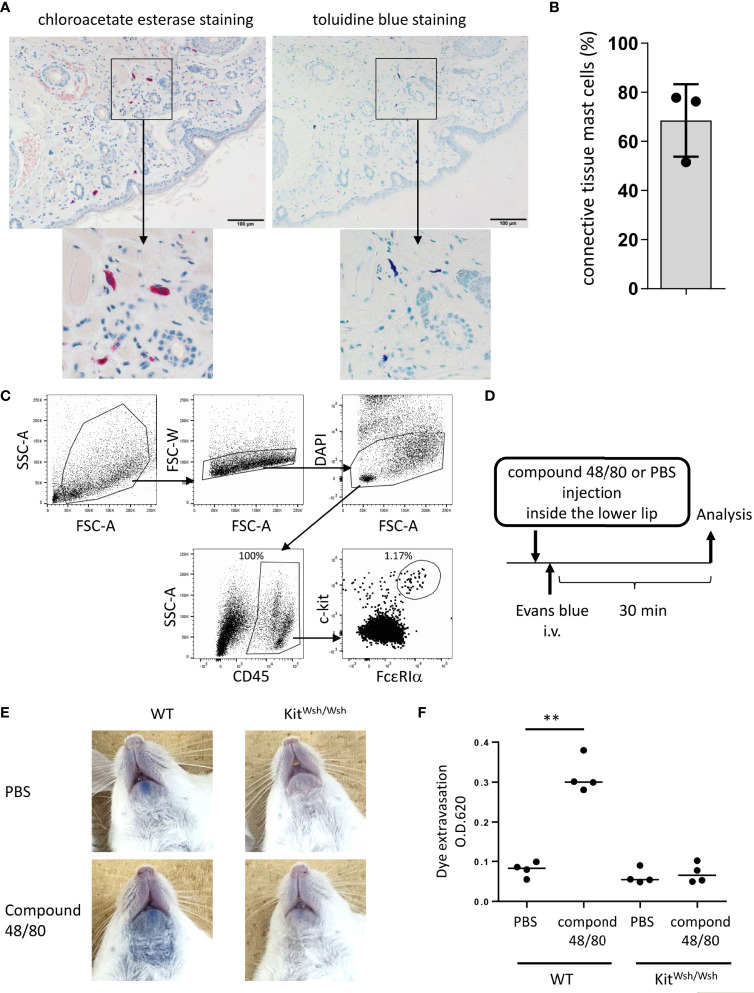
Development of murine model of pseudo-allergic reaction in the oral cavity. **(A, B)** Chloroacetate esterase staining or toluidine blue staining of serial tissue sections in the inside of the lower lip from Balb/c mice (n = 3) under steady conditions. **(A)** Chloroacetate esterase-stained (left panel) or toluidine blue-stained (right panel) mast cells are seen. **(B)** The percentage of both chloroacetate esterase- and toluidine blue-stained mast cells among chloroacetate esterase-stained mast cells. The total count was 59, 33, or 45 mast cells in serial tissue sections from three mice. Data are representative of two independent experiments. **(C)** Surface expression levels of FcεRIα and c-Kit among CD45^+^ hematopoietic cells in the inside tissue of the lower lip from Balb/c mice under steady conditions. FcεRIα^+^c-Kit^+^ mast cell populations (1.17%) among CD45^+^ cells (100%) were seen. Data are representative of three independent experiments. **(D)** A schematic representation of murine models of compound 48/80-induced pseudo-allergic reaction in oral cavities. **(E)** Injection of compound 48/80 inside the lower lip caused dye extravasation in the skin from WT mice but not from *Kit^W-sh^/^W-sh^* mice. Representative pictures of the neck skin from WT mice injected with compound 48/80 (left/lower) or PBS (left/upper) and from *Kit^W-sh^/^W-sh^* mice injected with compound 48/80 (right/lower) or PBS (right/upper) **(F)** Quantification of the Evans blue dye that extravasated into the neck skin from WT or *Kit^W-sh^/^W-sh^* mice injected with compound 48/80 or PBS. n =4 per group; data are presented as mean ± SD. Data are representative of two independent experiments. Brown-Forsythe and Welch ANOVA with Dunnett T3 multiple comparisons. **P < 0.01.

**Figure 2:**
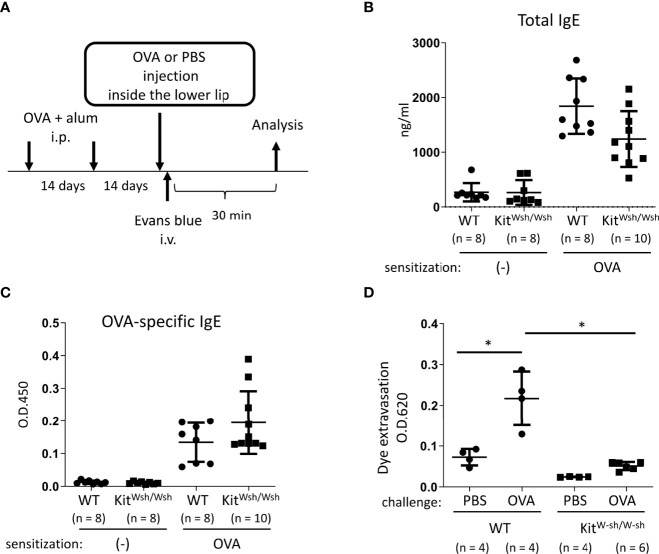
Development of murine model of IgE- and mast cell-mediated allergic reaction in oral cavities. **(A)** A schematic representation of murine models of OVA-induced, IgE- and mast cell-dependent allergic reaction in oral cavities. **(B, C)** Serum levels of total IgE **(B)** and OVA-specific IgE **(C)** from WT mice (n = 8) or *Kit^W-sh^/^W-sh^* mice (n = 10) intraperitoneally injected with OVA plus alum twice at a 2-week interval or from non-sensitized WT mice (n = 8) or *Kit^W-sh^/^W-sh^* mice (n = 8). **(D)** Quantification of the Evans blue dye that extravasated into the neck skin from OVA-sensitized WT mice stimulated by OVA (n = 4) or PBS (n = 4) or *Kit^W-sh^/^W-sh^* mice stimulated by OVA (n = 6) or PBS (n = 4). Data are presented as mean ± SD. Data are representative of two independent experiments. Brown-Forsythe and Welch ANOVA with Dunnett T3 multiple comparisons. *P < 0.05.

**Figure 3:**
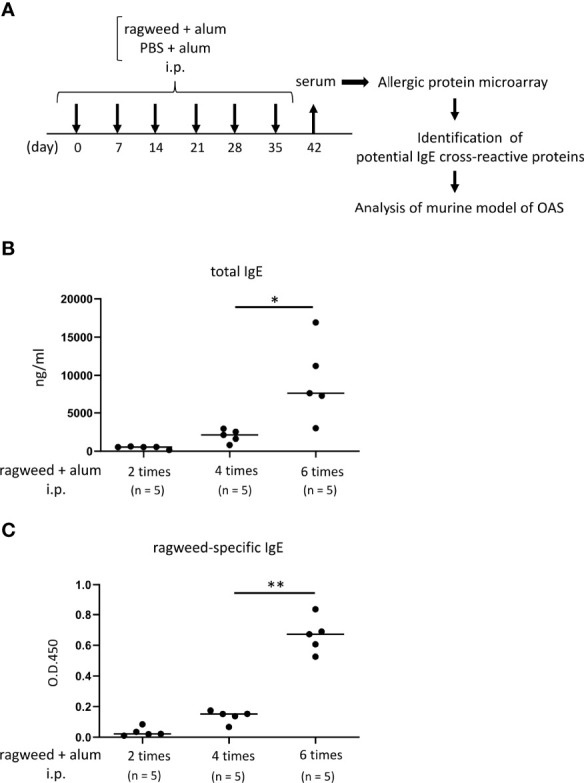
Identification of foods that may cross-react with ragweed pollen by using murine model of sensitization with ragweed pollen and allergenic protein microarray technology. **(A)** Allergenic protein microarray analysis was performed using serum from mice given intraperitoneal administration of ragweed pollen plus alum or PBS plus alum as a control, before analysis of murine model of OAS. **(B, C)** Balb/c mice (n = 5) were intraperitoneally injected with ragweed pollen plus alum six times at a 1- week interval. Serum levels of total IgE **(B)** and ragweed-specific IgE **(C)** from Balb/c mice after two, four or six times of intraperitoneal injection of ragweed pollen plus alum. Data are presented as mean ± SD. Data are representative of two independent experiments. Brown-Forsythe and Welch ANOVA with Dunnett T3 multiple comparisons. *P < 0.05; **P < 0.01.

**Figure 4:**
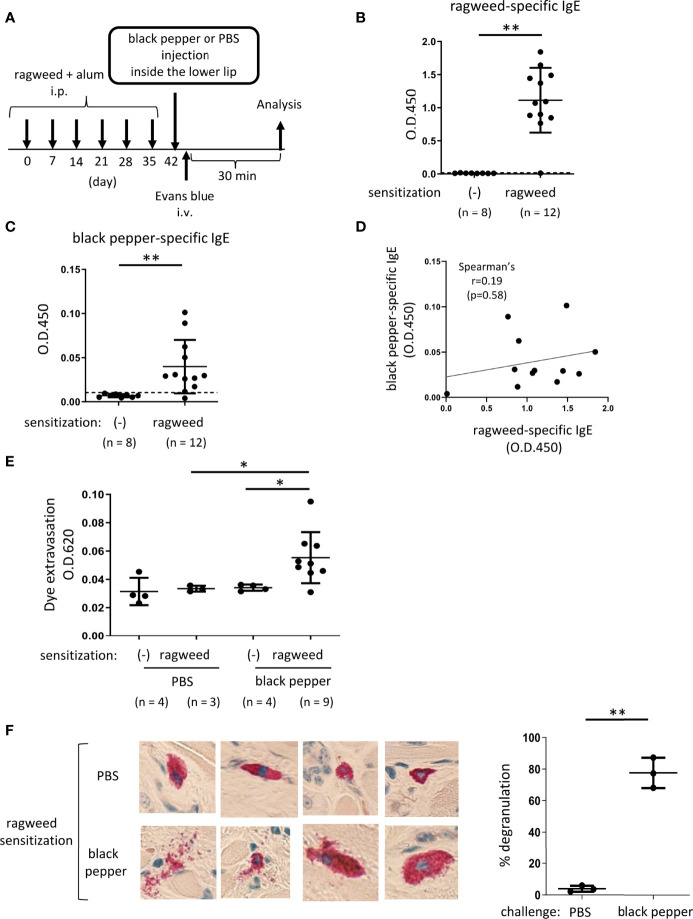
Ragweed pollen cross-reacts with black pepper in murine model of OAS. **(A)** A schematic representation of murine model of OAS. WT mice intraperitoneally sensitized with ragweed pollen before stimulation with black pepper or PBS as a control. **(B-D)** Serum levels of ragweed-specific **(B)** and black pepper-specific IgE **(C)** from ragweed-sensitized mice at day 42 (n = 12) or non-sensitized control mice (n = 8). **(B and C)** A positivity threshold (mean + 2SD in non-sensitized mice) is shown as a dotted line. Data are presented as mean ± SD. Data are representative of two independent experiments. Welch’s *t*-test. **P < 0.01. **(D)** No correlation between serum levels of ragweed-specific IgE and black pepper-specific IgE from ragweed-sensitized mice (n =12). Spearman’s correlation test. **(E)** Quantification of the Evans blue dye that extravasated into the neck skin from ragweed-sensitized mice stimulated by black pepper extract (n = 9) or PBS (n = 3) or from non-sensitized control mice stimulated by black pepper extract (n = 4) or PBS (n = 4). Data are presented as mean ± standard deviation. Data are representative of two independent experiments. Brown-Forsythe and Welch ANOVA with Dunnett T3 multiple comparisons. *P < 0.05. **(F)** Representative images of images of chloroacetate esterase-stained mast cells in tissue sections in the inside of the lower lip from ragweed-sensitized mice stimulated by black pepper extract (left/lower panel) or PBS (left/upper panel). Percentages of degranulated mast cells were shown (right). n = 3 per each group. Data are presented as mean ± SD. Data are representative of two independent experiments. Welch’s *t*-test. **P < 0.01.

**Figure 5:**
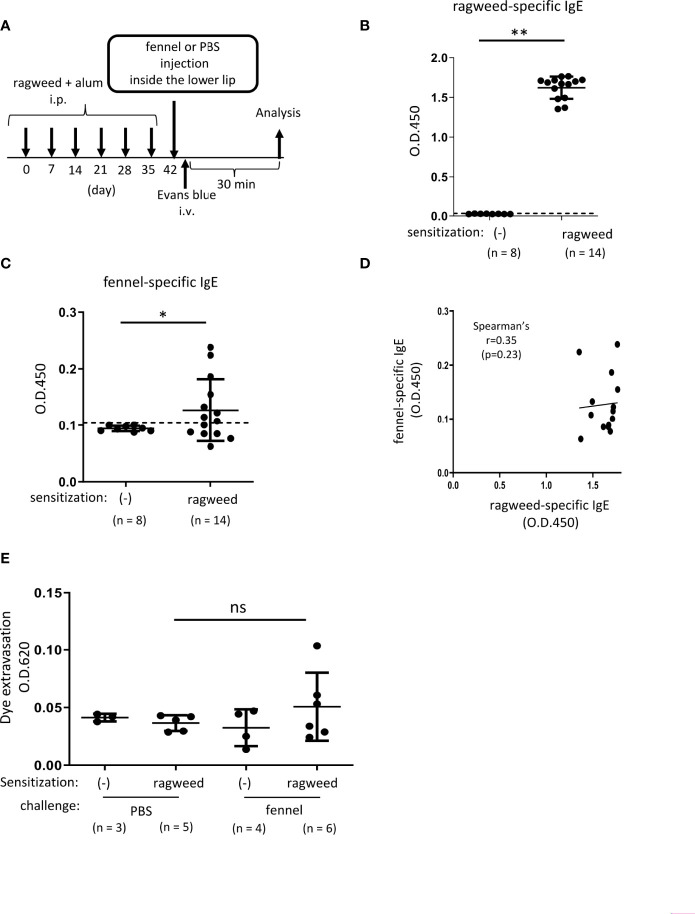
Stimulation with fennel extracts did not significantly induce OAS in mice intraperitoneally sensitized with ragweed pollen in murine model. **(A)** A schematic representation of murine model of OAS. WT mice intraperitoneally sensitized with ragweed pollen before stimulation with fennel or PBS as a control. **(B-D)** Serum levels of ragweed-specific IgE **(B)** and fennel-specific IgE **(C)** from mice intraperitoneally sensitized with ragweed pollen at day 42 (n = 14) or non-sensitized control mice (n = 8). **(B, C)** A positivity threshold (mean + 2SD in non-sensitized mice) is shown as a dotted line. Data are expressed as mean ± SD. Data are representative of two independent experiments. Welch’s *t*-test. **P < 0.01. **(D)** No correlation between serum levels of ragweed-specific IgE and fennel-specific IgE from ragweed-sensitized mice (n = 14). Spearman’s correlation test. **(E)** Quantification of the Evans blue dye that extravasated into the neck skin from ragweed-sensitized WT mice stimulated by fennel extract (n = 6) or PBS (n = 5) or from non-sensitized control mice stimulated by fennel extract (n = 4) or PBS (n = 3). Data are expressed as mean ± SD. Data are representative of two independent experiments. Brown-Forsythe and Welch ANOVA with Dunnett T3 multiple comparisons. ns, not significant.

**Figure 6:**
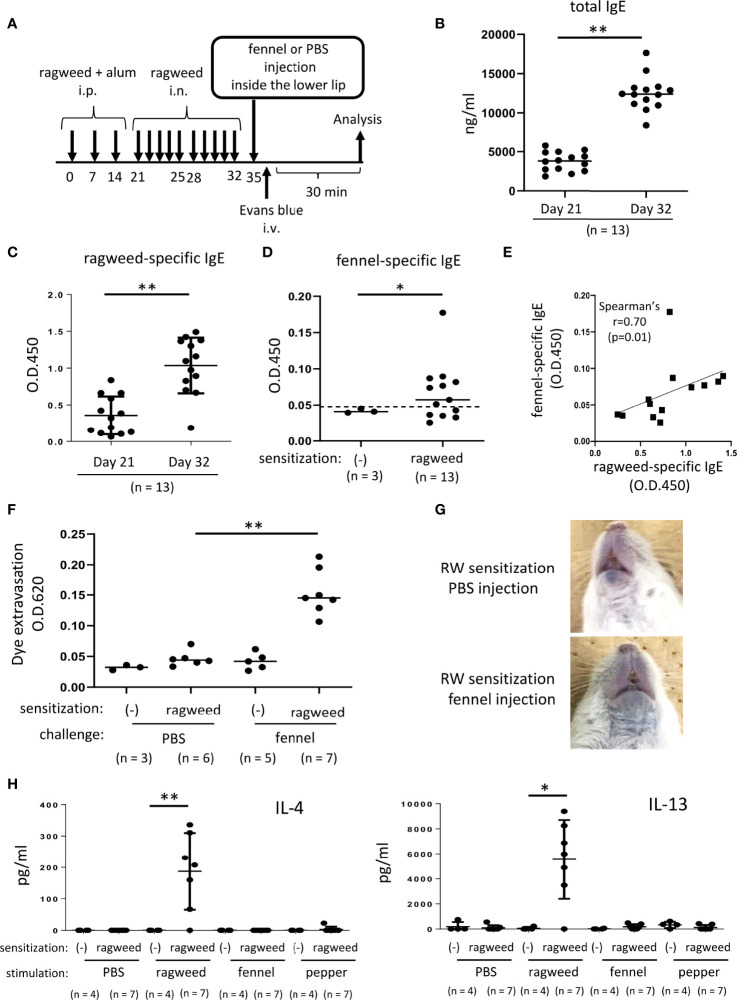
Stimulation with fennel extracts induced mast cell-dependent OAS in mice intraperitoneally and intranasally sensitized with ragweed pollen. **(A)** A schematic representation of murine model of OAS. WT mice intraperitoneally and intranasally sensitized with ragweed pollen before stimulation with fennel or PBS as a control. **(B-D)** Serum levels of total IgE **(B)** and ragweed-specific IgE **(C)** from ragweed-sensitized mice (n = 13) at day 21 and at day 32. **(D)** Serum levels of fennel-specific IgE from ragweed-sensitized mice (n = 13) at day 32 or from non-sensitized control mice (n = 3). A positivity threshold (mean + 2SD in non-sensitized mice) is shown as a dotted line. Data are expressed as mean ± SD. Data are representative of two independent experiments. Welch’s *t*-test. *P < 0.05; **P < 0.01. **(E)** A weak positive correlation between serum levels of ragweed-specific IgE and fennel-specific IgE from ragweed-sensitized mice (n = 13). Spearman’s correlation test. **(F)** Quantification of the Evans blue dye that extravasated into the neck skin from ragweed-sensitized mice stimulated by fennel extract (n = 7) or PBS (n = 6) or from non-sensitized control mice stimulated by fennel extract (n = 5) or PBS (n = 3). Data are expressed as mean ± SD. Data are representative of two independent experiments. Brown-Forsythe and Welch ANOVA with Dunnett T3 multiple comparisons. **P < 0.01. **(G)** Representative pictures of the neck skin from ragweed-sensitized mice stimulated by fennel extracts (lower panel) or PBS (upper panel). Data are representative of two independent experiments. **(H)** Levels of IL-4 (left) and IL-13 (right) in the culture supernatants from cervical LN cells from ragweed-sensitized mice (n = 7) or non-sensitized control mice (n = 4) in response to ragweed pollen, fennel, or black pepper extract. Data are expressed as mean ± SD. Data are representative of two independent experiments. Brown-Forsythe and Welch ANOVA with Dunnett T3 multiple comparisons. *P < 0.05; **P < 0.01.

**Figure 7:**
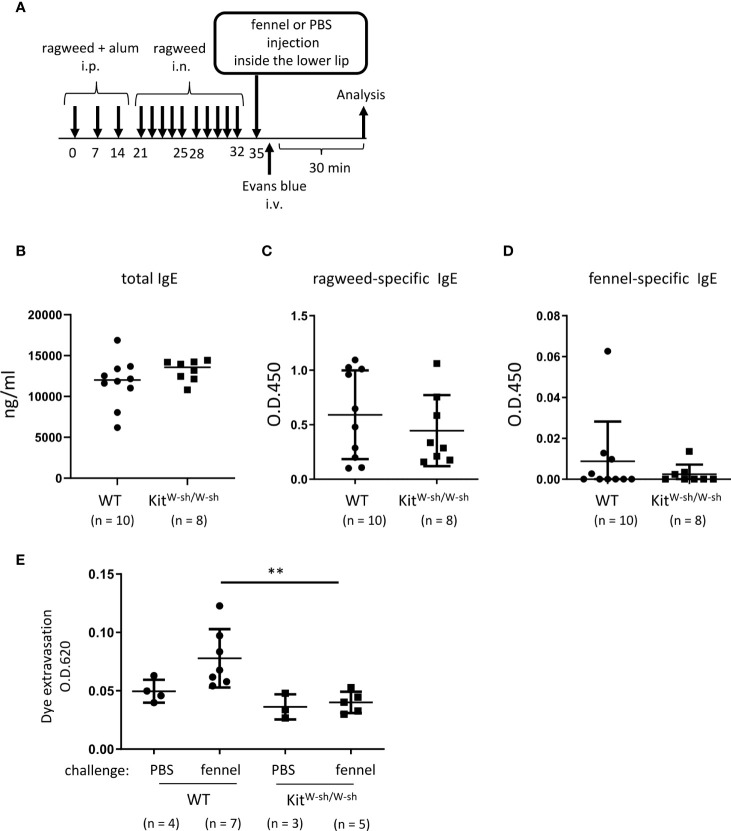
Stimulation with fennel extracts induced mast cell-dependent OAS in mice intraperitoneally and intranasally sensitized with ragweed pollen. **(A)** A schematic representation of murine model of OAS. WT or *Kit^W-sh^/^W-sh^* mice intraperitoneally and intranasally sensitized with ragweed pollen before stimulation with fennel or PBS as a control. **(B-D)** Serum levels of total IgE **(B)**, ragweed-specific IgE **(C)**, or fennel-specific IgE **(D)** from WT (n = 10) or *Kit^W-sh^/^W-sh^* (n = 8) mice at day 35. **(E)** Quantification of the Evans blue dye that extravasated into the neck skin from ragweed-sensitized WT mice stimulated by fennel extracts (n = 7) or PBS (n = 4) or from *Kit^W-sh^/^W-sh^* mice stimulated by fennel extracts (n = 5) or PBS (n = 3). Data are expressed as mean ± SD. Data are representative of two independent experiments. **(B-D)** Welch’s *t*-test. **(E)** Brown-Forsythe and Welch ANOVA with Dunnett T3 multiple comparisons. **P < 0.01.

Data are expressed as mean ± standard deviation (SD). Brown-Forsythe and Welch analysis of variance (ANOVA) with Dunnett T3 multiple comparisons was used in **Figures 1F**, **2B**–**D**, **3B**, **C**, **4E**, **5E**, **6F**, **H**, and **7E**. Welch’s *t*-test was used in **Figures 4B**, **C**, **F**, **5B**, **C**, **6B**–**D**, **7B**-**D**. Spearman’s correlation test was used in **Figures 4D**, **5D**, and **6E**. *p < 0.05 or **p < 0.01 was considered statistically significant.

## Results

### Development of murine model of pseudo-allergic reaction in oral cavities

We focused on lip angioedema, one of the most common symptoms of OAS, in the development of murine models of OAS. Histological analysis showed that chloroacetate esterase-stained mast cells were distributed in tissues from inside the lower lip of Balb/c mice (**Figure 1A**) and approximately 70% of the total mast cells were both toluidine blue- and chloroacetate esterase-stained mast cells, corresponding to connective tissue mast cells (**Figures 1A**, **B**) (5, 18, 35, 36, 45). Flow cytometric analysis showed that FcεRI^+^c-Kit^+^ mast cells resided in the tissues of the lower lip (**Figure 1C**). Because compound 48/80 is known to degranulate connective tissue mast cells through Mas-related G protein-coupled receptor b2 in mice (41, 46), we investigated whether stimulation with compound 48/80 leads to mast cell degranulation in murine oral cavities. To this end, the mice were intravenously injected with the Evans blue dye immediately after the injection of compound 48/80 or PBS as a control inside the lower lip (**Figure 1D**). The results showed that injection with compound 48/80, but not with PBS, induced apparent dye extravasation in the skin of the neck (**Figure 1E**). In sharp contrast, dye extravasation was not observed in mast cell-deficient *Kit^W-sh/W-sh^* mice treated with compound 48/80 (**Figure 1E**). Consistent with this, measuring the amount of extravasated dye in the skin showed that an injection of compound 48/80 caused higher amounts of dye extravasation in WT mice as compared to that following PBS injection (**Figure 1F**). Comparably low amounts of dye extravasation were observed in compound 48/80- and PBS-stimulated *Kit^W-sh/W-sh^* mice (**Figure 1F**). Therefore, we developed murine model to evaluate oral pseudo-allergic reactions presumably due to mast cell degranulation.

### Development of murine models of IgE- and mast cell-mediated allergic reaction in oral cavities

Next, we developed murine model of IgE- and mast cell-mediated allergic reactions in the oral cavity. WT and *Kit^W-sh/W-sh^* mice were intraperitoneally injected with OVA plus alum adjuvant twice at 2-week intervals (**Figure 2A**). We found elevated levels of total IgE and OVA-specific IgE in the serum from immunized mice 2 weeks after the last injection of OVA plus alum. We confirmed that mast cell deficiency did not significantly influence the serum levels of OVA-specific IgE (**Figures 2B**, **C**). The mice were intravenously injected with the Evans blue dye immediately after injection of OVA or PBS as a control inside the lower lip (**Figure 2A**). Notably, the OVA injection induced apparent dye extravasation in the skin of OVA-sensitized WT mice, but not of OVA-sensitized *Kit^W-sh/W-sh^* mice, whereas PBS injection failed to do so in either mice groups. Consistently, the OVA-challenged WT mice exhibited higher amounts of extravasated dye in the skin than the OVA-challenged *Kit^W-sh/W-sh^* mice or PBS-challenged WT mice (**Figure 2D**). Thus, we developed murine model to evaluate oral allergic reactions, which appear to depend on IgE-driven mast cell degranulation.

### Identification of foods that may cross-react with ragweed pollen

To identify foods that may cross-react with ragweed pollen, we used murine model of sensitization with ragweed pollen in BALB/c mice as well as protein microarray technology (34). First, the mice were intraperitoneally injected with ragweed pollen plus alum at 1-week intervals (**Figure 3A**). We observed a gradual increase in the serum levels of total IgE and ragweed-specific IgE after repeated injections (**Figures 3B**, **C**). Serum levels of ragweed-specific IgE on day 42 after the sixth injection were significantly higher than those on day 28 after the fourth injection (**Figure 3C**). We then performed allergenic protein microarray analyses using serum from the mice after the sixth injection with ragweed plus alum or alum alone as a control. A total of 1178 types of crude protein extracts from a variety of plants, animals, processed foods, and microorganisms, including vegetables, fruits, fish, shellfish, meats, eggs, cheeses, yogurts, insects, ticks, parasites, bacteria, and fungi were examined to calculate the relative binding affinity to respective protein extracts of serum IgE from ragweed pollen-sensitized mice versus control mice. All top 22 protein extracts, which were highly bound by serum IgE from the ragweed pollen-sensitized mice, were derived from plants (**Table 1**). Among them, nine protein extracts, including those ranked first, were derived from ragweed, including *Ambrosia artemisiifoli* (common ragweed), *Ambrosia trifida* (giant ragweed), and *Ambrosia bidentata* (lanceleaf ragweed), a member of the *Asteraceae* family, demonstrating the reliability of this assay. The second- and third-ranked were derived from *Sambucus nigra*, a member of the *Adoxaceae* family, whereas those raked fourth and fourteenth were from fennel, a member of the *Apiaceae* family. *Piper nigrum* (Black peppercorn), *Salvia officinalis* (common sage), *Plantaho asiatica*, *Orthosiphon aristatus* (cumisctin), *Prunus campanulate*, *Ficus pumila*, *Capsicum annuum* (paprika), *Sambucus nigra*, and *Foeniculum vulgare* (fennel) were edible (**Table 1**). In any case, some of the above-described plants may include allergenic proteins that cross-react with ragweed pollen allergens.

**Table 1:** Relative binding intensity of serum IgE to the respective protein extracts.

	ID	Classification-1	Classification-2	Allergen	Intensity
1	000981	plant	*Asteraceae*	*Ambrosia artemisiifoli* (common ragweed)	2.64
2	001037	plant	*Adoxaceae*	*Sambucus nigra*	2.27
3	001017	plant	*Adoxaceae*	*Sambucus nigra*	2.23
4	000980	plant	*Apiaceae*	*Foeniculum vulgare* (fennel)	1.90
5	001042	plant	*Asteraceae*	*Ambrosia artemisiifolia* (common ragweed)	1.85
6	000005	plant	*Asteraceae*	*Ambrosia artemisiifolia* (common ragweed)	1.83
7	001022	plant	*Asteraceae*	*Ambrosia artemisiifolia* (common ragweed)	1.78
7	001025	plant	*Asteraceae*	*Ambrosia bidentata* (lanceleaf ragweed)	1.78
9	001045	plant	*Asteraceae*	*Ambrosia bidentata* (lanceleaf ragweed)	1.71
10	000389	plant	*Asteraceae*	*Ambrosia trifida* (giant ragweed)	1.67
10	000505	plant	*Calophyllaceae*	*Calophyllum inophyllum*	1.67
12	000390	plant	*Asteraceae*	*Ambrosia artemisiifolia (ragweed)*	1.58
13	000544	plant	*Piperaceae*	*Piper nigrum* (black peppercorn)	1.57
14	000849	plant	*Lamiaceae*	*Salvia officinalis* (common sage)	1.52
14	001000	plant	*Apiaceae*	*Foeniculum vulgare* (fennel)	1.52
16	000213	plant	*Plantaginaceae*	*Plantago asiatica*	1.40
17	000130	plant	*Lamiaceae*	*Orthosiphon aristatus* (cumisctin)	1.39
18	000225	plant	*Rosaceae*	*Cerasus campanulata*	1.38
18	000210	plant	*Moraceae*	*Ficus pumila*	1.38
18	000834	plant	*Solanaceae*	*Capsicum annuum* (paprika)	1.38
21	000967	plant	*Asteraceae*	*Ambrosia artemisiifolia* (ragweed)	1.35
21	000478	plant	*Malvaceae*	*Gossypium* spp. (cotton linters)	1.35

The relative binding intensity of serum IgE from ragweed-sensitized mice to the respective protein extracts (ID, Classification-1, Classification-2, and Allergen) was estimated by subtracting the IgE-binding intensity of serum from mice injected with alum alone from IgE-binding intensity of serum from mice injected with ragweed pollen plus alum.

### Ragweed pollen cross-reacts with black pepper in murine model of OAS

Based on the results of the protein microarray analyses, we investigated whether ragweed pollen cross-reacts with black pepper, which is frequently used as a cooking spice. WT mice, that had been intraperitoneally injected with ragweed pollen plus alum six times weekly, exhibited high levels of ragweed-specific IgE in the serum (**Figures 4A**, **B**). Intriguingly, significantly higher levels of black pepper-specific IgE in the serum were also observed in the same ragweed-sensitized mice than in the non-sensitized control mice (**Figure 4C**). There was no correlation between the serum levels of ragweed-specific IgE and black pepper-specific IgE (**Figure 4D**). The mice were intravenously injected with the Evans blue dye immediately after the injection of black pepper extract or PBS as a control inside the lower lip. The results showed that significantly higher amounts of dye extravasation were evident in ragweed-sensitized mice injected with black pepper extract but not with PBS (**Figure 4E**). In accordance with this, histological analyses of the inside of the lower lip revealed frequent distribution of degranulated mast cells in the former mice, but not in the latter mice (**Figure 4F**). It should be noted that the injection of black pepper extract or PBS did not induce apparent dye extravasation in the non-sensitized control mice (**Figure 4E**). Collectively, these results indicate that black pepper allergen crosslinked ragweed-specific IgE-bound FcεRI on mast cells, leading to mast cell degranulation inside the lower lip. Thus, we were able to provide evidence of IgE cross-reactivity between ragweed pollen and black pepper in murine model.

### Stimulation with fennel extracts did not significantly induce OAS in mice intraperitoneally sensitized with ragweed pollen in murine model

We then examined whether ragweed pollen cross-reacts with fennel, which is frequently used as a cooking spice. Similarly, we used WT mice that received intraperitoneal injections of ragweed pollen plus alum six times weekly (**Figure 5A**). The results showed that the serum levels of fennel-specific IgE as well as ragweed-specific IgE in ragweed pollen-sensitized mice were higher than those in non-sensitized control mice (**Figures 5B**, **C**). There was no correlation between the serum levels of ragweed-specific IgE and fennel-specific IgE (**Figure 5D**). Although intravenous injection of the Evans blue dye immediately after an injection of fennel extract inside the lower lip did not cause dye extravasation in non-sensitized control mice, similar injections of fennel extracts, but not of PBS, tended to increase the amount of dye extravasation in ragweed pollen-sensitized mice (**Figure 5E**).

### Stimulation with fennel extracts significantly induced OAS in mice intraperitoneally and intranasally sensitized with ragweed pollen in murine model

Since we did not find definite evidence for IgE cross-reactivity between ragweed pollen and fennel *in vivo*, we attempted to use a ragweed pollen-induced allergic rhinitis model (37–40). The mice were administered an intranasal injection of ragweed pollen for five consecutive days in the first and second weeks after intraperitoneal injection of ragweed pollen three times weekly (**Figure 6A**). The results showed that intranasal administration of ragweed pollen strongly elevated the serum levels of total IgE and ragweed-specific IgE in mice intraperitoneally injected with ragweed pollen (**Figures 6B**, **C**). In addition, serum levels of fennel-specific IgE were higher in mice intraperitoneally and intranasally injected with ragweed pollen on day 35 than in non-sensitized control mice. (**Figure 6D**). There was a weak positive correlation between the serum levels of ragweed-specific IgE and fennel-specific IgE (**Figure 6E**). Importantly, the intravenous injection of the Evans blue dye immediately after injection of fennel extract, but not of PBS, inside the lower lip induced significant amounts of dye extravasation in mice intraperitoneally and intranasally injected with ragweed pollen on day 35 (**Figures 6F**, **G**). When cervical LN cells from these ragweed pollen-administered mice were *ex vivo* stimulated with ragweed pollen, fennel, or black pepper extracts, we found a significant increase in IL-4 and IL13 levels in the culture supernatants of cervical LN cells in response to ragweed pollen, but not to black pepper or fennel extract (**Figure 6H**) (35).

### Stimulation with fennel extracts induced mast cell-dependent OAS in mice intraperitoneally and intranasally sensitized with ragweed pollen

We then used the same model in WT and *Kit^W-sh/W-sh^* mice to examine the role of mast cells in these responses (**Figure 7A**). The results showed that mast cell deficiency did not significantly influence serum levels of total IgE, ragweed-specific IgE, or fennel-specific IgE (**Figures 7B** **-** **D**). However, mast cell deficiency dampened dye extravasation following an injection of fennel extract inside the lower lip in mice intraperitoneally and intranasally injected with ragweed pollen, suggesting that these responses in the oral cavities were induced in an IgE- and mast cell-dependent manner (**Figure 7D**). Taken together, we were able to provide evidence of IgE cross-reactivity between ragweed pollen and fennel in murine model.

## Discussion

OAS/PFAS are frequently caused by IgE cross-reactivity between food and pollen allergens. Indeed, some combinations of cross-reactive pollen and food are well-known by allergologists. Although cross-reactive allergens have been identified by taking a history of patients with suspected OAS/PFAS, detecting allergen-specific IgE in the serum, or performing a skin prick test, unknown combinations of allergens with cross-reactivity may cause unexplained OAS/PFAS (6–17). In the present study, we developed a method to comprehensively identify potential cross-reactive allergens. Analysis of allergenic protein microarray identified several edible plants to which serum IgE from ragweed pollen-sensitized mice can strongly bind. The reliability of this assay was supported by the finding that nine out of 11 extracts derived from ragweed were included in the top 22 extracts that were highly bound by serum IgE from ragweed pollen-sensitized mice. In addition, we developed methods to evaluate the degree of IgE-mediated mast cell degranulation in oral cavities, reflecting the severity of OAS in murine model: immediately after an injection of OVA inside the lower lip in mice with high levels of serum OVA-specific IgE, an intravenous injection of the Evans blue dye induced apparent dye extravasation in the neck skin, of which amounts could be quantitated. Although oral rubbing after an oral challenge was reported to be a symptom of PFAS in murine model (47), the quantitation of extravasated dye seemed to be suitable for evaluating IgE cross-reactivity of allergens of interest *in vivo*. In addition, such murine model will be necessary for definite identification of IgE cross-reactive allergens inducing OAS/PFAS, because it is clinically known that IgE cross-reactive allergens do not necessarily cause OAS/PFAS even though IgE cross-reactivity is shown by serological tests (6–17). Accordingly, we were able to test whether an edible plant that potentially cross-reacts with ragweed pollen induces OAS in our murine model. Indeed, an injection of fennel or black pepper extract caused high amounts of dye extravasation in the neck skin of ragweed-sensitized mice but not of non-sensitized control mice. If such an injection induced apparent dye extravasation in control mice, the reaction would presumably be caused by IgE-independent increase in vascular permeability. In the case of an injection of fennel extract, mice intraperitoneally and intranasally injected with ragweed pollen, experiencing allergic rhinitis, exhibited stronger allergic responses in the oral cavity than mice intraperitoneally injected with ragweed pollen, suggesting that the former sensitization method is more appropriate for analyzing IgE cross-reactivity in murine model of OAS. In either case, mice were intraperitoneally administered ragweed pollen plus alum to strongly induce ragweed-specific IgE production. In addition, mice were challenged by injection of fennel or black pepper extract inside the lower lip to strongly induce mast cell degranulation in the oral cavity, where exaggerated allergic responses might have been caused. Therefore, our murine model seems useful to identify IgE cross-reactive allergens. However, these sensitization and challenge routes seem not suitable for analyzing the pathophysiology of OAS/PFAS. For this end, we should use a different murine model in which mice are orally challenged with food extract after intranasal sensitization with pollen. In any case, we provided evidence of IgE cross-reactivity of ragweed pollen with black pepper or fennel.

Intriguingly, we found Th2 cytokine production in LN cells from ragweed pollen-sensitized mice only in response to ragweed pollen extract, and not in response to fennel or black pepper extract, suggesting that T cell reactivity is not the same with ragweed pollen and fennel or black pepper (1, 16). However, it should be noted that the crude extracts of food or pollen contain various allergens with different quantities and stabilities, which may depend on their processing. Accordingly, the analysis of *ex vivo* Th2 responses of LN cells using recombinant allergenic protein available as well as crude extracts of food or pollen in murine model will be useful for understanding the pathogenesis of OAS/PFAS.

Considering that celery-mugwort-spice syndrome is known as PFAS, it may be reasonable to assume that ragweed pollen would cross-react with black pepper, a member of the *Piperaceae* family, and fennel, a member of the *Apiaceae* family (11, 17, 19–22, 48, 49). Our results are also supported by those of a previous report that fennel possibly cross-reacts with mugwort, paprika, ragweed, or black pepper in a case with occupational rhinitis and asthma (50, 51). Nevertheless, our experimental results regarding IgE cross-reactivity will be informative for clinical allergologists and patients with OAS/PFAS. Profilin Amb a 8 or lipid transfer protein Amb a 6, which is known as a ragweed pollen allergen, might be involved in the ragweed-fennel-black pepper association (11, 26, 27, 29, 33). Otherwise, biochemical techniques will be required to identify a specific allergen included in ragweed pollen and clarify how ragweed pollen cross-reacts with fennel or black pepper (6–17). One limitation of this study is that the allergens in ragweed, fennel, or black pepper extracts used were not molecularly defined.

In this allergenic protein microarray analysis, crude protein extracts from various foods, including edible plants, were coated on microarray plates; however, allergens contained in such foods can be quantitatively or qualitatively influenced by cultivar, climates, or chemical treatments. The stability of each allergen can be affected to varying degrees by protein extraction procedures (52). This may explain why melon and banana were not identified as food extract that highly cross-react with serum IgE from ragweed pollen-sensitized mice, notwithstanding that the ragweed-melon-banana association was previously reported as PFAS (24–26, 28). Alternatively, the difference of immune responses between humans and mice may be taken into consideration. In any case, microarray analysis using each recombinant allergenic molecule will lead to more comprehensive and direct identification of IgE cross-reactive molecules (1, 52). Importantly, our analysis using protein microarray technology and murine model of OAS will complement the traditional analysis of serum from patients with OAS/PFAS patients to clarify the molecular mechanisms of the syndrome caused by IgE cross-reactivity. The allergen

In conclusion, we have developed a method to identify cross-reactive allergens using murine model of sensitization to specific allergens and OAS as well as protein microarray technology, which will help understand the pathogenesis of OAS/PFAS.

## Data availability statement

The original contributions presented in the study are included in the article/Supplementary Material. Further inquiries can be directed to the corresponding author.

## Ethics statement

This study was reviewed and approved by the institutional review boards of Juntendo University (approval numbers 2020128 and 2020136)

## Author contributions

AnK performed all the experiments and participated in writing the manuscript. TA, HW, and KT assisted with the *in vitro* experiments. AyK, TI, AM, HY, MK, RY, and NN assisted with the *in vivo* experiments. TS, HO, and KO analyzed the data. KI and JK conceived the project, analyzed the data, and actively participated in manuscript writing. All authors contributed to the article and approved the submitted version.

## Funding

This study was supported by JSPS KAKENHI Grant Numbers 17H04217 and 20H03721 and by a Grant-in-Aid for Special Research in Subsidies for ordinary expenses of private schools from The Promotion and Mutual Aid Corporation for Private Schools of Japan.

## Acknowledgments

We thank the Laboratory of Morphology and Image Analysis, Research Support Center, Juntendo University Graduate School of Medicine for technical assistance.

## Conflict of interest

The authors declare that the research was conducted in the absence of any commercial or financial relationships that could be constructed as a potential conflict of interest.

## Publisher’s note

All claims expressed in this article are solely those of the authors and do not necessarily represent those of their affiliated organizations, or those of the publisher, the editors and the reviewers. Any product that may be evaluated in this article, or claim that may be made by its manufacturer, is not guaranteed or endorsed by the publisher.

## Abbreviations

ANOVA, analysis of variance; APC, allophycocyanin; Cy7, cyanine 7; ELISA, enzyme-linked immunosorbent assay; IL, interleukin; LN, lymph node; OAS, oral allergy syndrome; OVA, ovalbumin; PBS, phosphate-buffered saline; PE, phycoerythrin; PFAS, pollen food allergy syndrome; SD, standard deviation; WT, wild-type.
